# Exploring the experiences of changes to support access to primary health care services and the impact on the quality and safety of care for homeless people during the COVID-19 pandemic: a study protocol for a qualitative mixed methods approach

**DOI:** 10.1186/s12939-020-01364-4

**Published:** 2021-01-10

**Authors:** Kelly Howells, Martin Burrows, Mat Amp, Rachel Brennan, Wan-Ley Yeung, Shaun Jackson, Joanne Dickinson, Julie Draper, Stephen Campbell, Darren Ashcroft, Tom Blakeman, Caroline Sanders

**Affiliations:** 1 NIHR Greater Manchester Patient Safety Translational Research Centre, 6th Floor Williamson Building, Oxford Road, Manchester, M13 9PL, Centre for Primary Care, The University of Manchester, Manchester, UK; 2 Groundswell, London, UK; 3 Salford Primary Care Together, Salford, UK; 4 Urban Medical Practice, Manchester, UK; 5 Bolton Homeless and Vulnerable Adults Service, Bolton, UK

**Keywords:** Primary care, Homelessness, Equality, Covid-19

## Abstract

**Background:**

Despite high level of health care need amongst people experiencing homelessness, poor access is a major concern. This is sometimes due to organisational and bureaucratic barriers, but also because they often feel stigmatised and treated badly when they do seek health care. The COVID-19 pandemic and the required social distancing measures have caused unprecedented disruption and change for the organisation of primary care, particularly for people experiencing homelessness. Against this backdrop there are many questions to address regarding whether the recent changes required to deliver services to people experiencing homelessness in the context of COVID-19 will help to address or compound problems in accessing care and inequalities in health outcomes.

**Methods:**

An action led and participatory research methodology will be employed to address the study objectives. Interviews with people experiencing homelessness were will be conducted by a researcher with lived experience of homelessness. Researchers with lived experience are able to engage with vulnerable communities in an empathetic, non-judgemental way as their shared experience promotes a sense of trust and integrity, which in turn encourages participation in research and may help people speak more openly about their experience. The experiences of health professionals and stakeholders delivering and facilitating care for people experiencing homelessness during the pandemic will also be explored.

**Discussion:**

It is important to explore whether recent changes to the delivery of primary care in response to the COVID-19 pandemic compromise the safety of people experiencing homelessness and exacerbate health inequalities. This could have implications for how primary healthcare is delivered to those experiencing homelessness not only for the duration of the pandemic but in the future.

## Background

The COVID-19 pandemic and the required social distancing measures have caused unprecedented disruption and change for the organisation of primary care. Telephone consultations and remote ‘virtual’ consultations via video technology (such as accurx, Attend Anywhere, Vision, TPP and EMIS) have been rolled out at speed to prevent the spread of COVID-19 [1]. Pre-COVID, there had been mounting policy pressure to implement digital innovations to increase staff capacity and access to primary care services [2]. However, there have also been concerns about a so-called ‘digital divide’ with certain population groups being left behind and excluded from potential benefits of technologies that could then exacerbate existing health inequalities [3, 4]. Previous research to evaluate remote care and particularly telemedicine consultations has also highlighted patient safety concerns that stem from communication changes that fundamentally alter interpersonal interaction influencing ability to pick up on body language and non-verbal cues, or sensory aspects (e.g. signs of inflammation and infection [1, 5, 6]. The psycho-social aspects of professional-patient relationships (e.g. communication and trust) are also considered to be a crucial underpinning for patient safety within primary care contexts, especially amongst people with long-term or sensitive health conditions where stigma is often experienced (e.g. sexual health or mental health problems), or when people feel they may be judged for poor health behaviours (e.g. smoking, alcohol and drug use) [7–9]. These issues are particularly relevant when considering access and experiences of primary care for homeless people in the context of rapid changes in service provision currently happening as a means of enabling continued access for this marginalised population group.

People experiencing homelessness are a vulnerable group and are at increased risk of complications from the virus, whether this be directly from catching the virus, or indirectly due to the impact the pandemic has on their ability to access healthcare for their existing health conditions. These issues are crucial given the high rates of physical and mental health problems experienced by homeless people, and especially for rough sleepers [10, 11]. People experiencing homelessness commonly have a history of childhood trauma and experience mental health problems (80%) and high rates of drug and alcohol problems. Physical health problems are also much more common than the general population including poor oral health, chronic pain and other long-term conditions [12]. Chronic homelessness is commonly associated tri-morbidity [13, 14] used to refer to the combination of physical ill-health with mental ill-health and drug or alcohol misuse and gives rise to complex needs. People experiencing homelessness are also at increased risk of premature mortality with average age of death being 47 for a man and 43 for a woman [15]. There have also been increasing numbers of deaths, up 24% in 5 years with 597 deaths recorded on the streets or in temporary accommodation in England and Wales in 2017 [16].

The literature on the health and healthcare experiences of homeless people indicates a number of major patient safety concerns. Despite high level of health care need,, poor access is a major concern as oeople experiencing homelessness often fail to get appropriate healthcare, sometimes due to organisational and bureaucratic barriers, but also because they often feel stigmatised and treated badly when they do seek health care [17]. This highlights problems with continuity of care and care transitions, and discharge back to the streets has been highlighted as a major impact on prolonged suffering and preventable deaths. Against thisbackdrop, there are many questions to address regarding whether recent changes required to deliver services to people experiencing homelessness in the context of COVID-19 will help to address or compound problems in accessing care and inequalities in health outcomes.

Research has demonstrated that telephone consultations can improve access to care for patients and reduce workload for staff, and some research has pointed to enhanced benefits of video consultations particularly when patients are particularly anxious [18]. Whilst, there have been concerns that video consultations can be disruptive to organisational routines, systematic reviews indicate they can provide safe and high quality care for ‘clinically appropriate’ patients but most evidence has been based on people with stable chronic conditions [19, 20]. Video consultations were found to mirror face to face consultations in terms of consultation length and style, particularly if the patient and practitioner already have an established relationship. However Greenhalgh et al. [1, 19] argue that such an evolvement in consultation style may be challenging to embed long term due to practical barriers, such as funding, and individual factors, such as risk averse practitioners [21]. System changes, particularly rapid changes, may create new challenges to delivering healthcare and there may be unintended negative consequences of such changes for patient access to and experience of care [21, 22]. Practitioners have raised concerns that the safety and quality of medical advice could be compromised by limited physical contact, which could lead to complaints regarding clinical negligence [1]. There are hence questions as to whether the risks of digital innovations in healthcare outweigh the benefits [22]. However in the context of a health crisis, primary care practitioners have had no choice but to implement such innovations quickly to enable access to care. Video consultations are now available in 97% of practices, covering 98% of the population. Prior to COVID-19 only 5% of practices were live with video consultations. By the end of March 2020, it was approximately 66% [1]. Greenhalgh et al. [1] suggest that within the context of COVID-19, video and telephone consultations will be appropriate for some patients, such as those managing chronic conditions or experiencing symptoms of COVID-19, but they are unlikely to be appropriate replacements for seriously ill patients or for those who are not experienced using digital technologies.

Much of the existing literature on the use of digital health innovations for people experiencing homelessness has originated from research in the US [23] and mobile text messaging based interventions appear as most acceptable and feasible. However, there is a lack of evidence on the use or barriers to use of digital technologies amongst marginalised groups for supporting navigation of health and social care systems [24]. There is also no evidence as to whether remote video and telephone consultations may increase access to healthcare for people experiencing homelessness or merely exacerbate existing inequalities. A recent review of digital remote care interventions for vulnerable groups with chronic disease has also highlighted the need to consider contextual factors and particular support needs for enabling use of any such interventions, and particularly the role of social networks [25].

However, people experiencing homelessness are heterogeneous and some may adapt to remote consultations easier than others, particularly if they have access to the digital resources needed. There are also particular challenges for meeting needs for complex and sensitive physical health conditions (such as infected skin and foot ulcers and wounds, or sexual health care), and mental health problems and changes to the prescribing and monitoring of medications to treat drug and alcohol addiction.

There is a paucity of research into the benefits of video consultations in primary care, particularly for improving the access and engagement of marginalised groups, and the current changes already underway as localities across Greater Manchester attempt to make changes to support healthcare access presents the opportunity to research what can be viewed as a ‘natural experiment’ [1]. These changes provide important context for research experiences, needs and potential interventions to tackle patient safety concerns associated with homelessness.

The setting and context for the research within a rapidly changing system indicates the need to respond to diverse changes that will necessarily be influenced by multiple complexities and interdependencies regarding the ways in which changes and technologies are implemented within distinct localities. This requires an approach to evaluation that responds to this complexity and is able to document and feedback evidence as in a ‘learning health system’ approach. Such an approach is characterised by a participatory culture that acknowledges there is no simple replicable way of implementing change in a complex system and requires flexible systems to respond to local context [19]. Qualitative social science methods are important to support continuous learning based on understanding of processes and mechanisms associated with interaction and experiences of testing new innovations for improving quality and safety of care that may work very different within distinct contexts [22]. Additionally, the quantitative analysis of available data gathered routinely within systems can support ongoing learning and evidence based decision-making. This forms the rationale for this qualitative case study approach to evaluating how primary healthcare providers have responded to providing care for people experiencing homelessness across Greater Manchester (GM).

### The aims of the study

The study aims to evaluate the experience and impact of organisational and technology changes (such as facilitation of remote consultations), in response to the COVID-19 pandemic on access to healthcare and the quality and safety of care for people experiencing homelessness.

1. The specific objectives are: To explore the experiences of people experiencing homelessness in accessing and using health services during the COVID-19 pandemic
2. Explore how experiences of care influence the quality and safety of care (e.g. communication, continuity, support for self-management, use of medications, diagnosis and treatment).
3. To explore the perspectives of health professionals and key stakeholders regarding patient safety concerns in delivering healthcare for people experiencing homelessness during the COVID-19 Pandemic.

### Study design

As outlined above, we draw on a learning health systems approach which is consistent with a participatory and action research approach that has informed the initial stages of our work. This approach has been widely used for conducting community or service based research, including amongst people experiencing homelessness and service providers (e.g. [26–29]. Action research and participatory methods refer to styles of research that emphasise collaboration and democratic working between multiple partners to bring about change [30, 31]. Such approaches are characterised by periods of action and reflection [32]. This cyclical and participatory nature is also reflected in recent methodologies such as Experience Based Design (EBD [33];) and Experience Based Co-Design (EBCD [34]; commonly used for design of informatics and healthcare interventions, as well as quality improvement within healthcare.

It is important the study design not only takes into account individual experiences of access to primary care during the pandemic but also consider the organisational and practice context in which these changes are implemented. It is likely GP practices will vary as to how they facilitate access to care for people experiencing homelessness during the pandemic. For example, some may facilitate this process by providing homeless hostels and hotels with smart technology, but for those living in temporary accommodation, there may be more of a reliance on peer support or voluntary organisations. As the demographics of local homeless populations may differ, e.g. some areas may have more street homeless, sex workers or refugees, general practices may approach delivering care to these more vulnerable patients in different ways. To evaluate these complex changes it is therefore important to compare and contrast experiences across GP practice sites, including associated pharmacies, in GM and to do this we suggest a case study approach.

A case study approach is used to generate an in-depth understanding of ‘complex issues in its real-life’ context [35]. The case study approach investigates individual level experience but also takes into account organisational context and the wider social and policy context. The collective case study approach involves sampling multiple cases so that contextual similarities and differences can be taken into account [35]. We will aim to sample 4 primary care sites e.g. 2 specialist homeless general practices and 2 additional practices in different localities and associated hotels, hostels and pharmacies linked to those practices.

We will adopt a qualitative approach to understanding and evaluating each case study. Flexible semi structured interviews will be conducted with health professionals, other stakeholders (such as hostel workers, volunteer organisations), peer mentors and people with lived experience of homelessness. While qualitative research is common in health services research, interviews generally take place face to face within the health care context [36]. Due to on-going social distancing rules, this will not be possible and all interviews will be conducted over the phone. Face to face interviews are generally preferred in qualitative research as it helps the researcher establish rapport and observe body language and social cues, which may change the direction of the interview. However it has well established that telephone interviews also have their advantages as they are more anonymous in nature and this may help some people discuss more sensitive issues. Rapport can also be established via verbal listening cues so that the participant is aware the researcher is listening [36].

## Qualitative study: phase 1 – understanding the perspectives and experiences of health professionals and stakeholders

### Sampling and recruitment

#### Case study sites

We aim to recruit 3–4 case study sites across GM and evaluate their approach to delivering primary care for people experiencing homelessness during the pandemic. We will work with three specialist homeless general practices in Manchester and Salford who have already started working with some local hostels and pharmacies to facilitate remote healthcare during the pandemic. We will also work with a specialist homeless clinical services based in Bolton.

GPs already collaborating on the study will discuss the study with their colleagues and collect email addresses of anyone willing to be contacted. Potential participants will then be sent a participant information sheet and consent form via email by the University team. If they respond to the email, a time and date will be arranged to contact them via phone. The study and information sheet will be discussed over the phone and if they are still happy to participate, consent will be taken with an encrypted audio recorder. This will be saved in a separate file to the research data. All interviews will be audio recorded with an encrypted recorder and we anticipate the interviews to last between 30 and 60 min maximum.

#### Semi-structured interviews

We will conduct audio recorded telephone interviews with GPs, nurses, pharmacists and local partners, such as hostel and charity workers, about their perceptions and experiences of providing and facilitating primary care for people experiencing homelessness since the beginning on the pandemic. We aim to interview 5–10 health professionals and stakeholders from each case study site.

Stakeholders who provide healthcare and support to people experiencing homelessness will be asked about their perceptions of issues that fall under the umbrella of patient safety, and more specifically about their perspectives on key issues regarding patient safety for people experiencing homelessness since the pandemic. We will prompt for general safety concerns as perceived by the participant followed by prompts informed by the patient safety literature.

The topic guide will be developed and with stakeholder involvement (e.g. Urban Medical Practice) and tested prior to formal data collection. Questions will be broad but also informed by the existing literature on homeless health care provision and the key patient safety issues associated with this patient population. Questioning in follow up interviews will also adapt according to prior data collection to utilise findings from the initial interview. The interviews will be audio-recorded and professionally transcribed via a University of Manchester approved transcription service.

#### Audio-diaries

GPs and nurses will be asked to keep an audio diary of their experiences of delivering remote consultations. They will be asked to record their views on how the consultation went well/badly, and also any patient safety concerns they may have. This method of data collection has been chosen to minimise the influence of the research team on what health professionals deem important in delivering healthcare remotely for people experiencing homelessness. We plan to ask health professionals to keep an audio diary for a minimum of 1 month (patient names will not be recorded) and this audio diary will be used to gain insight into any concerns practitioners may experience as they happen. Practitioners will be provided with an encrypted audio recorder and their reflections will be thematically coded in NVIVO.

The results of the audio-diary combined with the concerns and experiences of people experiencing homelessness and other stakeholders will be used to feedback to GPs on how services can be improved. Follow up interviews at the end of study will take place with healthcare professionals and stakeholders to determine how things have progressed and changed during the pandemic.

#### Follow up interviews with health professionals, including audio- diaries

Health professionals in general practices recruited to the study will be asked to participate in a follow up interview approximately 3–6 months after the baseline interview. During this period, some of the GPs and nurses recruited to the study will be asked to keep a recorded ‘diary’ of their experiences and any reflections they have on remote consultations. Encrypted audio-recorders will be provided by the University of Manchester for the recordings. The recorders will be collected by the University of Manchester researcher approximately 1 month after the start date. Participants would need special software on their computers to download the audio recordings themselves and NHS firewall will not allow this software to be uploaded, which is why we will collect the audio recordings rather than ask participants to download the recordings themselves. Healthcare professionals involved will be asked to record no patient personal data and to describe any cases or observations anonymously. Information regarding the recordings will be provided in a SOP for the health professionals involved. GPs often do this as part of their GP training and are therefore used to recording their concerns in this way. These recorded reflections will be listened to and notes taken (by the UOM research team) and used in the follow up interviews to prompt discussion. It will not be compulsory to keep an audio diary to participate in the follow-up interviews. Following the follow up interview, these audio-files will be deleted. Any notes will be saved with a pseudonymised ID and saved on RDS.

Follow up interviews will also be conducted over the phone. A separate information sheet will be provided and consent will be retaken. This will not be guided by an interview schedule as interviews will be based on participant’s previous interviews and also audio-diaries (if applicable). Follow up interviews will last between 30 and 60 min.

#### Follow up interviews with stakeholders

As above, we will gain consent to re-contact stakeholders for a follow up interview. If they agree, they will be re-contacted and sent a follow up interview information sheet via email. Consent will be retaken and interviews will be conducted over the phone. Stakeholders will not be required to keep an audio diary. All interviews will be conducted with an encrypted audio recorder. Interviews will be unstructured as based on previous interviews with participants. Follow up interviews will last between 30 and 60 min.

### Recruiting participants with lived experience of homelessness

#### Sample

We aim for a purposive sample to represent a diverse range of participants according to age, gender, ethnicity, types of health problems (including long-term mental and physical health problems). We will also include people who are either currently homeless or people with recent experience of homelessness during the pandemic. By homelessness we are referring to people who are street homeless, live in temporary accommodation or are ‘sofa surfing’. This definition also includes homeless families.

Gatekeepers will approach eligible participants about the study and give them a participant information sheet so they can make an informed choice about participating and if they are interested, they will be asked for consent to pass their phone number to a researcher from Groundswell. Due to social distancing measures, all interviews will take place over the phone. If they do not have a telephone, the Gatekeeper will arrange a time for the Groundswell researcher to call the hostel/key worker to speak to the participant. When the researcher from Groundswell calls the participant, they will discuss the content of the information and if they are still interested, audio- recorded consent will be taken over the phone. Posters will also be displayed in hostels so that individuals can contact Groundswell and express their interest directly.

The mixed sample will allow for recent reflections on patient safety issues experienced by those who are no longer homeless who may have greater capacity to compare and contrast receiving health care in the varying scenarios as well as those experiencing any patient safety issues as a result of their current homelessness. In total we will aim to recruit 5–10 people with lived experience of homelessness per site.

The University of Manchester is working with a homeless charity called Groundswell that will also conduct interviews with people experiencing homelessness. These interviews will not be conducted by staff at the University of Manchester, but the University will work with Groundswell to co-analyse the transcripts. All interviews conducted by Groundswell will be conducted with an encrypted audio recorder. All interviews, including follow up interviews will last approximately 30 min.

##### Semi- structured interviews

Interviews with participants experiencing homelessness will be undertaken by a researcher from Groundswell who has lived experience of homelessness. Demographic data (age, gender and ethnic origin) will be taken. Participants will be given an ID number and this ID number will also be used to save the audio-recordings and transcripts. Consent forms will be saved under an anonymous ID so it cannot be linked to research data. The pseudonymised ID number will also be recorded on the participant’s contact information spreadsheet. Contact information will be deleted at the end of the study.

Interviews will also be audio-recorded with an encrypted recorder and topics will include questions about previous and current health problems and health care needs, as well as who they seek help from when they have health problems and what has changed during the pandemic. Prompts will be used to elicit specific examples of perceived safety issues and their views about the quality and safety of care.

Specific questions about their experience of remote consultations and how it compares to their previous experience of receiving care will also be asked. Participants will also be asked if they would be willing to participate in a follow up interview 3–6 months after the initial interview to discuss how their experiences have changed.

### Follow up interviews

We also aim to conduct some follow up interviews with people experiencing homelessness if possible regarding their experiences over the next 3–6 months. This interview will not be compulsory and may not always be possible due to the transient nature of this population. Participants who consent to the initial interview and also consent to be re-contacted, will be telephoned/emailed to discuss the follow up interview. If possible, a follow up PIS will be emailed to participants and read out over the phone. Consent will also be retaken. Interviews will be unstructured as based on previous interviews with participants.

### Data analysis

Interviews will be recorded digitally and transcribed ensuring that the data are anonymised. Initial notes and reflections will be made by researchers (both UoM and Groundswell) immediately following interviews to reflect on the processes, key issues raised in discussion, and any contextual issues that would not be reflected in transcripts. The qualitative data will be analysed using a framework method. This method produces a matrix of summarised data and provides a structure to analyse and summarise data within key themes. It is particularly suited to informing intervention design as well as enabling analysis effectively within teams of multiple researchers, and is highly suited to policy related research [37, 38]. The approach allows for analysis according to predefined themes and themes that emerge more inductively from the data [37]. Our initial analytical framework will be structured by some of the main aspects of experience reflected in the literature on homelessness and patient safety. This framework will be used as a starting point for coding initial transcripts, and the framework will be extended as new codes emerge inductively from the data. For these processes the Research Associate and the core research team based at University of Manchester will work closely with the Research Lead and Peer Researchers from Groundswell (MB and team) to do all stages of coding and thematic analysis. Members of the UoM team and the Groundswell team will conduct initial reading and coding of the transcripts and will chart and summarise these within the framework structure. The team will hold regular meetings in order to discuss emerging themes, and to develop and refine the final themes. This process will continue until no new themes emerge. The findings from this initial round of focus groups and interviews will feed into development of a number of possible ‘solutions’ in the form of potential interventions for discussion at follow-up workshops in phase 2.

### Patient and Public Involvement & Engagement

The Groundswell charity is a lead partner organisation for this project and will be co-producing this work with the UoM team. Groundswell will work with Shelter in Manchester in order to establish a public involvement advisory group made up of people with lived experience of homelessness who can meet at regular intervals throughout the development and conduct of the research. They will advise on key stages of the research and all information for participants, as well as providing input in developing appropriate material for dissemination of the research.

#### Challenges and limitations

Definitions of homelessness are varied and include people who are living in temporary accommodation and those who are ‘hidden homeless’, such as people who may be temporarily staying with friends or family or feel obliged to live with another person due to domestic violence. It may be more challenging to recruit the ‘hidden’ homeless people to participate in the research as by definition they are often not visibly homeless. However, we will endeavour to capture a wide range of diverse experiences by engaging with local stakeholders, such as hostels, day centres and outreach workers to act as gatekeepers to recruit people experiencing homelessness to the study. This will include charities that specialise in supporting women and sex workers, who are often more likely to meet the definition of ‘hidden homeless.

Peer-led research is associated strongly with social change, inclusivity and breaking down the traditional hierarchies between researchers and participants [39]. The approach is deemed effective in recruiting vulnerable groups to participate in research and producing research that is ‘meaningful’ as the rapport established results in ‘honest’, in-depth data [39, 40]. However, the inherent subjective nature of this approach has resulted in debates as to whether peer-produced research can be deemed valid and objective as peer-researchers can lack detachment from the research issue as their prior experiences and views may bias the data collection and analysis [41, 42]. Others have also raised concerns as to whether peer researchers have the specific skill set required to produce robust qualitative research data [43]. We acknowledge these potential issues but to ensure objective, valid research data is produced, Groundswell have developed a peer research programme to ensure their researchers have the skills and knowledge required to conduct sensitive and objective qualitative interviews. The research training includes a focus on ‘reflexivity’ and the importance of asking neutral, non-leading questions. To ensure the interviews are conducted in this way, all research interviews will be reviewed by the core research team and once transcribed, feedback will be provided to the peer researcher to highlight any issues to ensure future interviews are more objective and to increase reflexivity. Any part of the interview that has clearly been ‘led’ will not be included in the analysis. All interviews will be co-analysed by The University of Manchester and Groundswell to minimise interpretation bias. Each transcript will be coded by the main researchers (KH, MA, MB and CS) and they will meet regularly to discuss the analysis to increase the validity of the research findings.

## Discussion

COVID-19 and the required social distancing regulations has accelerated the digital-care revolution. While evidence suggests remote consultations may increase access to care for some individuals, there is concern that the ‘digital-divide’ will exacerbate health inequalities for vulnerable social groups. Research shows that people experiencing homelessness are already less likely to access healthcare due to a number of socio-economic and experiential factors, but there is limited evidence as to whether digital interventions could increase access to healthcare for this population. The aim of this study is to explore whether the recent changes to delivering primary care in a COVID-19 context will address or exacerbate helth inequalities for those experiencing homelessness. As the drive to deliver primary healthcare remotely is likely to continue post-COVID-19, the findings will help policy makers and local care providers develop strategies to ensure that vulnerable patient groups, such as the homeless, have equal access to care.

## Data Availability

No datasets are currently available for this current study.

## Abbreviations

UOM: University of Manchester
NHS: National Health Service
